# Global productivity and research trends of colorectal carcinoma: A scientometric analysis of studies published between 1980 and 2021

**DOI:** 10.1097/MD.0000000000033037

**Published:** 2023-02-22

**Authors:** İbrahim Tayfun Şahiner, Çetin Altunal

**Affiliations:** a Department of General Surgery, Hitit University School of Medicine, Çorum, Turkey; b Private Clinic, İstanbul, Turkey.

**Keywords:** bibliometric analysis, colon cancer, colorectal cancer, rectal cancer, trends

## Abstract

Although colorectal cancer (CRC) is a serious cause of death and has a significant impact on patients’ quality of life and incidence rate of CRC has increased among the younger populations, bibliometric research of CRC has not been conducted yet. To perform a comprehensive analysis of scientific publications on CRC using various statistical and bibliometric techniques. Publications on CRC published between 1980 and 2021 were downloaded from the Web of Science database and analyzed using statistical methods. The trending topics, collaborations among countries, and citation relationships were analyzed using bibliometric network visualization mapping. The number of articles to be probably published in the next 5 years was estimated using the exponential smoothing estimator. The Spearman’s correlation rank correlation coefficient was used to analyze the correlations among the variables. A total of 122,717 publications were found in the fields of oncology, gastroenterology, hepatology, and surgery. Of the published articles, 64,774 publications were research articles. The top five countries that contributed the most to the literature were the USA (16,604; 25.6%), China (10,567; 16.3%), Japan (7932; 12.2%), the UK (5009; 7.1%), and Italy (4287; 6.6%). The most prolific author, institution, and journal in the field of CRC were Zhang Y (n = 331), University of Texas System (n = 1646), and Diseases of the Colon and Rectum (n = 2090), respectively. The most influential journal based on the average number of citations received per article was CA-A Cancer Journal for Clinicians (citations per article; 286). There was a significant positive correlation between the number of articles produced by the countries on CRC and gross domestic product and human development index (*r* = 0.726, *P* < .001; *r* = 0.658, *P* < .001, respectively). Additionally, a significant moderate correlation of CRC was found with gross domestic product per capita (*r* = 0.711, *P* < .001). Keywords like overall survival, neoadjuvant chemoradiotherapy, locally advanced rectal cancer, robotic surgery, anastomotic leakage, chemoradiotherapy, metastatic colorectal cancer, *KRAS*, meta-analysis, colorectal surgery, and laparoscopic surgery were studied.

## 1. Introduction

Colorectal cancer (CRC) is a type of gastrointestinal malignancy originating from either the colon or rectum. Although the malignancy can be simply defined as colon or rectal cancers depending on their origin, they are often merged because of the several similar biological and clinical features.^[1]^ Adenocarcinoma is the most common colorectal malignancy (up to 95% of cases), followed by carcinoid tumors, gastrointestinal stromal tumors, lymphomas, and sarcomas.^[2]^ CRC is a complex disease that arises from the accumulation of genetic alterations in the key regulatory genes and pathways, including the RAS-MAPK, Wnt and P13K pathways. *KRAS*, neuroblastoma RAS viral oncogene homolog, and *BRAF* genes.^[3,4]^ Subsequently, it became clear that genetic mutations play a partial role in the colorectal carcinogenesis. Epigenetic variations in cancer-related genes and noncoding RNAs are believed to be strongly involved in cancer initiation and progression.^[3,4]^

In a large number of patients, CRC may remain silent for a long time until it grows and spreads substantially, adversely impacting the prognosis. In symptomatic patients, particularly in patients with advanced stage of cancer, the disease may cause changes in the bowel motility (e.g., diarrhea or constipation), occult or evident colorectal bleeding, abdominal discomfort, cramping, unexplainable weight loss, weakness, and fatigue.^[1]^ The leading risk factors for CRC include family history, physical inactivity, overweight and obesity, large intake of alcoholic beverages, precancerous conditions, tall stature, high consumption of red or processed meat, and modest intake of dairy products and foods containing whole grains or dietary fiber, avoiding which makes this disease potentially preventable.^[1,5,6]^ More than 50% CRC cases and deaths are attributable to modifiable risk factors and a substantial proportion of the cases could be further prevented through screening and surveillance.^[6]^ The incidence and mortality rates of CRC also substantially vary by geographical differences, race, and ethnicity. The sex disparity in CRC cases also varies with the age of the patients.^[6]^

In 2020, approximately 19.3 million cancer cases were diagnosed and about 10 million cancer deaths were reported worldwide. Female breast cancer has surpassed lung cancer as the most commonly diagnosed cancer, with an estimated 2.3 million new cases (11.7%), followed by lung (11.4%), colorectal (10.0%), prostate (7.3%), and stomach (5.6%) cancers. Lung cancer remained the leading cause of death due to cancer with an estimated 1.8 million deaths (18%) followed by colorectal (9.4%), liver (8.3%), stomach (7.7%), and female breast (6.9%) cancer cases. The incidence rates of CRC were found to be 29 and 20 cases per 100,000 men and women, respectively, in countries with high/very high human development index (HDI) versus 7.4 and 5.4 cases per 100,000 men and women, respectively, in countries with low/medium HDI.^[7]^ In general, CRC ranks third in terms of incidence rate but is second in the terms of mortality. There is a significant variation in the incidence rates of colon cancer in different regions of the world. The highest incidence rates were observed in Europe, Australia/New Zealand, Northern America, Hungary, and Norway, while the lowest incidence rates were seen in Africa and South Central Asia^[7]^ CRC is the second most common cause of cancer-related deaths in the United States.^[5,6]^ According to the latest report of the American Cancer Association, there are about 147,950 new cases of CRC in the United States in 2020, including 104,610 and 43,340 cases of colon cancer and rectal cancer, respectively.^[5,6]^

Colorectal cancer can be considered as a marker of socioeconomic development, as the incidence rate of CRC tends to rise uniformly with increasing HDI in the developing countries.^[7,8]^ The increasing incidence rate in the formerly low-risk and lower HDI countries probably reflects the effect of changes in lifestyle factors that are associated with increasing the colorectal cancer risk and suggests the importance of strengthening preventive measures and conducting appropriate screening programs especially in countries undergoing major social and economic transition.^[1,7]^ The accelerated decline since the 2000s is chiefly attributed to the increased colonoscopy screening and removal of precursor lesions.^[6,7,9]^ Despite consistent improvements in screening strategies and the development of effective treatment methods, 5-year survival rate for advance stages of cancer is still not promising.^[4,10]^ Although the overall incidence and mortality rates of CRC are continuously declining, this progress is increasingly confined to the older age groups.^[6]^

Bibliometrics is the technique to analyze the scientific publications using various statistical methods.^[11,12]^ The increasing number of publications has led to an increasing number of bibliometric studies in the various fields of medicine.^[11–13]^ Although CRC is a major cause of death with an increasing incidence rate in young age groups and has a significant impact on patients’ quality of life, a bibliometric research has not been conducted for CRC. This study aimed to perform a holistic analysis of the scientific publications on CRC published between 1980 and 2021 using various statistical and bibliometric methods.

## 2. Methods

### 2.1. Research strategy

Literature review was performed using the Web of Science Core Collection (WoS by Clarivate Analytics) database. The search was conducted in the period of 1980 to 2021 (access date: March 15, 2022). Publications were screened using different keywords related to CRC that yielded the list of all publications containing CRC-related keywords in their titles. Repeatability codes for researchers to access similar documents are as follows (search results may vary by date of access): (title = (“colorectal cancer*”) or title = (“bowel cancer*”) or title = (“colon cancer*”) or title = (“rectal cancer*”) or title = (“colorectal carcinoma*”) or title = (“bowel carcinoma*”) or title = (“colon carcinoma*”) or title = (“rectal carcinoma*”) or title = (“colorectal adenocarcinoma*”) or title = (“bowel adenocarcinoma*”) or title = (“colon adenocarcinoma*”) or title = (“rectal adenocarcinoma*”) or title = (“colectomy*”) or title = (“bowel resection”) or title = (“rectum resection*”) or title = (“colon resection*”) or title = (“anterior resection*”)).

### 2.2. Statistical analysis

The number of publications in the coming years was estimated by observing the past publication trends using the exponential smoothing estimator with seasonal correction run in the Microsoft Office Excel program. Bibliometric network visualizations and citation analysis were performed using the VOSviewer (Version 1.6.16, Leiden University’s Center for Science and Technology Studies) software package.^[14]^ The website (https://app.datawrapper.de) was used to draw the world map. Statistical analyses were performed using Statistical Package for the Social Sciences (Version: 22.0, SPSS Inc., Chicago, IL, License: Hitit University) software package. The data was checked for normality of distribution using the Kolmogorov–Smirnov test. The association between global scientific productivity on CRC and some economic and development indicators of countries was investigated by analyzing the correlation between the number of articles produced by countries and Gross Domestic Product (GDP), Gross Domestic Product per capita (GDP per capita), and HDI using the Spearman’s rank correlation coefficient as the data was non-normally distributed (data were obtained from the World Bank.^[15]^) Statistical significance was set at a *P* < .05.

## 3. Results

### 3.1. Overview of the included publications

The literature review in the WoS database yielded 176,364 publications in all research fields related to CRC published between 1980 and 2021. Of these, 122,717 publications in the fields of oncology, gastroenterology, hepatology, and surgery were included in the study. These publications were divided into articles (64,774; 52.7%), meeting abstracts (40,782; 33.2%), review articles (5904; 4.8%), proceedings papers (3784; 3%), and letters (3759; 3%). The remaining publication were grouped into other types of publications (editorial materials, corrections, news items, book chapters, notes, early access, retracted publications, retractions, corrections, additions, discussions, books, book reviews, reprints, data papers, expression of concern, publication with expression of concern, bibliographies, biographical items). Publications categorized as articles (n = 64,774) were included in the bibliometric analyses. It was found that 97% (n = 62,856) articles were published in English and the remaining articles were published in other languages, such German (n = 858), French (n = 562), Spanish (n = 225), Russian (n = 102), Italian (n = 50), Korean (n = 49), Polish (n = 46), Turkish (n = 12), Portuguese (n = 6), Chinese (n = 2), Danish (n = 2), Dutch (n = 2), and Slovenian (n = 2). Almost all of the articles were indexed in Science Citation Index Expanded (SCI-Expanded; n = 61,208; 94.4%) and Emerging Sources Citation (3149; 4.8%) indexes. The remaining few studies were indexed in Conference Proceedings Citation Index-Science, Social Sciences Citation Index, Book Citation Index-Science, and Conference Proceedings Citation Index-Social Science and Humanities.

### 3.2. Publication trends by year

**Figure 1.**
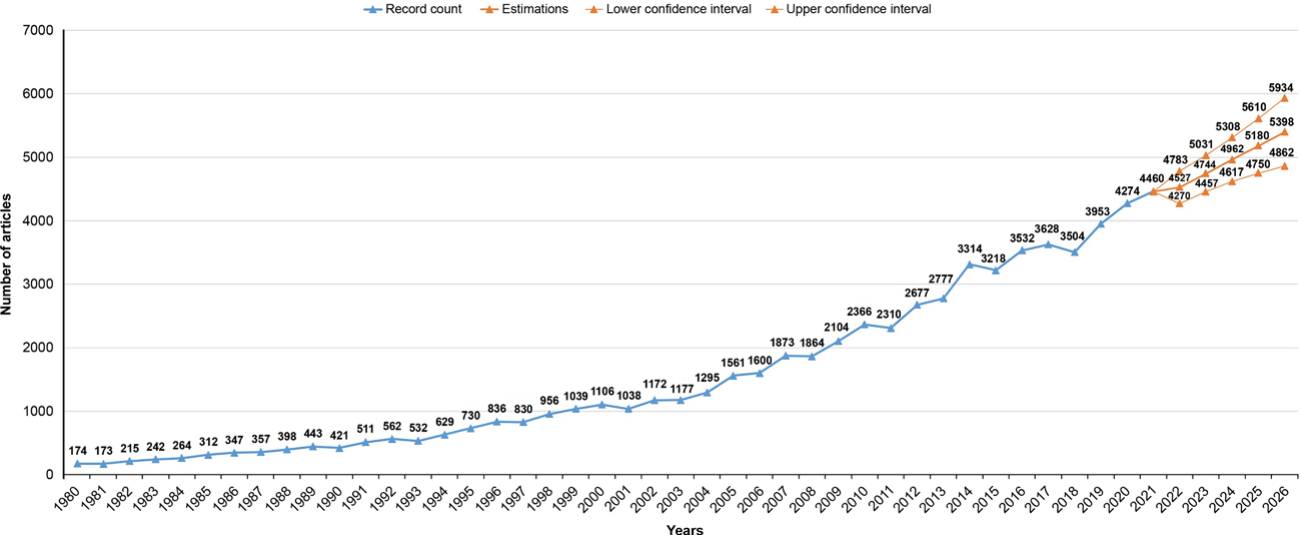
Bar graph showing the distribution of articles published on colorectal cancer in different years and the estimated number of articles for upcoming 5 years.

The number of published articles in different years is shown in Figure 1. The results of the exponential smoothing estimation model that were used to estimate the number of articles likely to be published 2022 onward are shown in Figure 1. The estimation model analysis revealed that approximately 4527 articles (95% confidence interval [CI]: 4270–4783) are likely to be published in 2022 and 5398 (95% CI: 4862–5934) articles will likely be published in 2026 (Fig. 1).

### 3.3. Active countries

**Figure 2.**
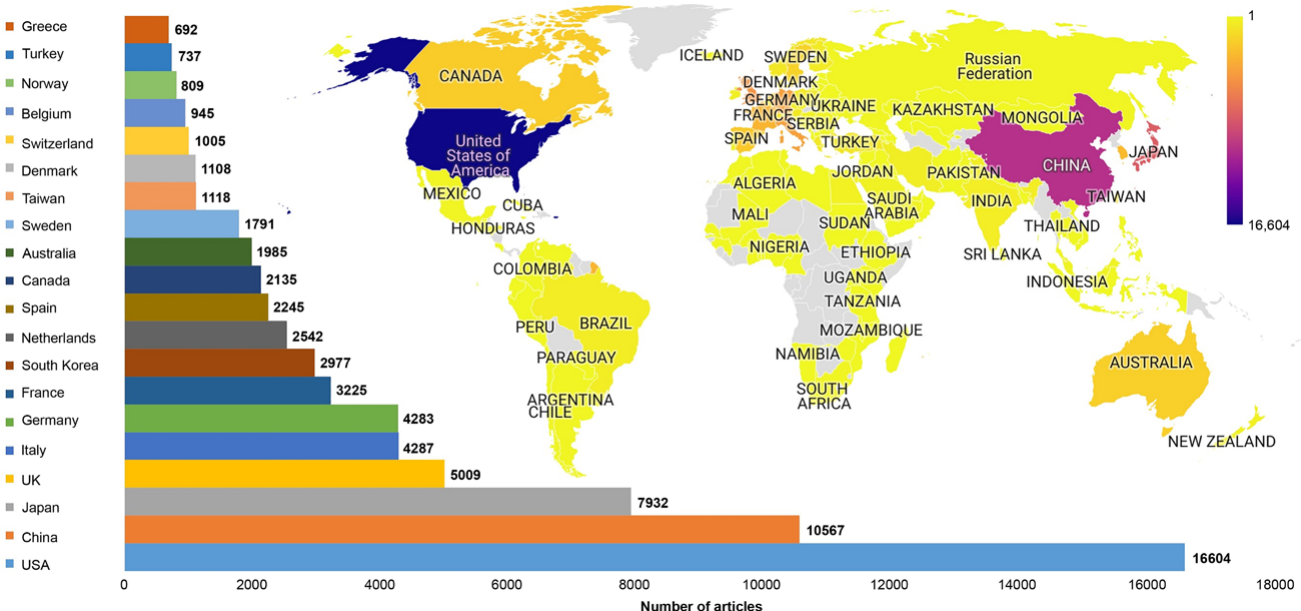
Global productivity world map showing the distribution of articles published on colorectal cancer by different countries. The bar chart shows the top 20 countries with the highest number of articles.

**Figure 3.**
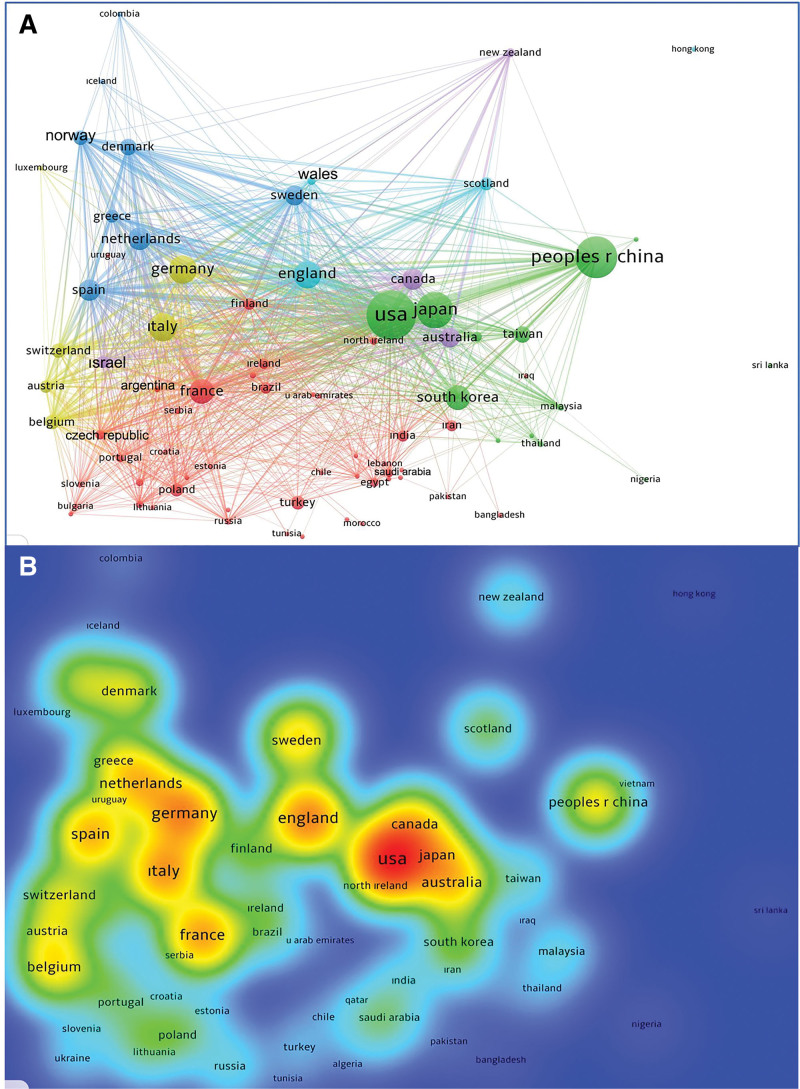
A. Network visualization map of cluster analysis showing international collaboration between countries for research on colorectal cancer. Different colors indicate different clusters. Bigger size of the circle indicates the higher number of articles published by the country. B. Density map showing the extent of international collaboration of countries for research on colorectal cancer. The strength of international collaboration score increases from blue to red (blue-green-yellow-red).

The number of articles produced by different countries is shown in Figure 2. The top 20 countries with the highest number of articles were the USA (16,604; 25.6%), China (10,567; 16.3%), Japan (7932; 12.2%), UK (5009; 7.1%), Italy (4287; 6.6%), Germany (4283; 6.6%), France (3225; 4.9%), South Korea (2977; 4.5%), Netherlands (2542; 3.9%), Spain (2245; 3.4%), Canada (2135; 3.2%), Australia (1985; 3.0%), Sweden (1791; 2.7%), Taiwan (1118; 1.7%), Denmark (1108; 1.7%), Switzerland (1005; 1.5%), Belgium (945; 1.4%), Norway (809; 1.2%), Turkey (737; 1.1%), and Greece (692; 1.0%). Of the 173 countries that had published articles on CRC, 79 countries that produced at least ten articles and had international collaboration among their authors were included in clustering analysis (Fig. 3A). The clustering analysis resulted in six different clusters relating to international collaboration (Colors for Cluster 1, red; Cluster 2, green; Cluster 3, blue; Cluster 4, yellow; Cluster 5, purple; Cluster 6, turquoise). Furthermore, the total link strength scores showing collaboration strength of 79 countries were calculated. The international collaboration density map was created on the basis of these scores is shown in Figure 3B. Top ten countries with the highest scores were the USA (8723), England (in the UK) (4838), Germany (4664), Italy (4216), France (3930), Spain (3521), Netherlands (3069), Australia (2504), Sweden (2387), and Belgium (2291).

### 3.4. Correlation analysis

There was a strongly significant positive correlation between the number of articles produced by countries on CRC and GDP and HDI (*r* = 0.726, *P <* .001; *r* = 0.658, *P <* .001, respectively); however, there was a moderately significant correlation of publication on CRC with GDP per capita (*r* = 0.711, *P <* .001).

### 3.5. Active authors

The top 15 most active authors on CRC were Zhang Y (n = 331), Wang L (n = 318), Watanabe T (n = 312), Li J (n = 300), Li Y (n = 297), Wang Y (n = 292), Fuchs CS (n = 284), Mori M (n = 282), Wang J (n = 252), Kim JH (n = 239), Watanabe M (n = 235), Brenner H (n = 232), Glimelius B (n = 231), Sugihara K (n = 229), and Kim NK (n = 224).

### 3.6. Active institutions

The top 20 institutions that produced the highest number of articles on CRC were the University of Texas System (n = 1646), Harvard University (n = 1642), University of California System (n = 1180), The University of Texas MD Anderson Cancer Center (n = 1168), Assistance Publique Hopitaux Paris (n = 1082), Institut National De La Sante Et De La Recherche Medicale (n = 1072), Unicancer (n = 1057), Mayo Clinic (n = 1037), Memorial Sloan Kettering Cancer Center (n = 959), Sun Yat Sen University (n = 937), Helmholtz Association (n = 850), National Institutes of Health (n = 835), National Cancer Center Japan (n = 834), German Cancer Research Center (n = 759), University of London (n = 753), National Cancer Institute (n = 751), Ruphts Karls University Heidelberg (n = 751), Karinsolka Institutet (n = 729), University of Toronto (n = 729), and University of Tokyo (n = 716).

### 3.7. Active journals

A total of 64,774 articles on CRC had been published in 836 different journals. The top 56 journals publishing 300 or more articles, the total number of citations received by the journals, and the average number of citations received per article are presented in Table 1.

**Table 1:** The 56 most active journals that have published more than 300 articles on colorectal cancer.

Journals	NA	C	AC	Journals	NA	C	AC
Diseases of the Colon and Rectum	2090	88,592	42.4	Gastroenterology	548	66,742	121.8
International Journal of Cancer	1533	74,373	48.5	Gut	542	46,534	85.9
Anticancer Research	1465	23,695	16.2	Cancers	535	2765	5.2
British Journal of Cancer	1410	70,625	50.1	Oncogene	524	30,753	58.7
International Journal of Colorectal Disease	1396	27,566	19.7	EJSO	474	9050	19.1
Cancer Research	1296	137,099	105.8	Carcinogenesis	462	26,057	56.4
Cancer	1157	67,563	58.4	World Journal of Surgery	456	13,443	29.5
Oncotarget	1120	27,903	24.9	Tumor Biology	455	8942	19.7
BMC Cancer	1116	26,740	24.0	International Journal of Radiation Oncology Biology Physics	442	21,511	48.7
World Journal of Gastroenterology	1092	29,399	26.9	American Journal of Surgery	422	12,378	29.3
Colorectal Disease	1057	19,031	18.0	Oncotargets and Therapy	410	4584	11.2
Oncology Reports	1048	20,299	19.4	Journal of Gastrointestinal Surgery	409	9034	22.1
Oncology Letters	972	9807	10.1	Current Colorectal Cancer Reports	375	1817	4.8
Annals of Surgical Oncology	944	37,643	39.9	World Journal of Surgical Oncology	367	3665	10.0
Clinical Cancer Research	939	65,200	69.4	Oncology	363	8557	23.6
Journal of Clinical Oncology	818	171,387	209.5	Digestive Diseases and Sciences	354	7132	20.1
International Journal of Oncology	745	18,541	24.9	Surgery Today	354	3814	10.8
European Journal of Cancer	744	31,420	42.2	Molecular Medicine Reports	344	4552	13.2
Surgical Endoscopy and Other Interventional Techniques	680	19,839	29.2	Nutrition and Cancer-An International Journal	339	8914	26.3
Journal of Surgical Oncology	676	14,996	22.2	Cancer Chemotherapy and Pharmacology	339	7339	21.6
British Journal of Surgery	669	52,040	77.8	Journal of Cancer Research and Clinical Oncology	334	6848	20.5
Cancer Letters	640	22,128	34.6	American Surgeon	333	4495	13.5
Cancer Epidemiology Biomarkers and Prevention	634	30,668	48.4	Asian Pacific Journal of Cancer Prevention	327	4551	13.9
Hepato-Gastroenterology	622	6978	11.2	Journal of Surgical Research	321	5340	16.6
Annals of Oncology	584	39,107	67.0	Scandinavian Journal of Gastroenterology	319	7241	22.7
Clinical Colorectal Cancer	560	8334	14.9	Journal of Experimental and Clinical Cancer Research	310	5859	18.9
Frontiers in Oncology	555	2787	5.0	International Journal of Clinical and Experimental Pathology	310	3388	10.9
Annals of Surgery	549	59,290	108.0	Cancer Science	307	8081	26.3

AC = average citation per document, C = number of citation, NA = number of articles.

### 3.8. Citation analysis

Of the 64,774 articles on CRC, the top 25 articles with the highest number of total citations are presented in Table 2. The last column of Table 2 indicates the average number of citations received by the articles per year.

**Table 2:** The top 25 most cited articles on colorectal cancer by total number of citations.

No	Article	Author	Journal	PY	TC	AC
1	Normalization of real-time quantitative reverse transcription-PCR data: a model-based variance estimation approach to identify genes suited for normalization, applied to bladder and colon cancer data sets	Andersen CL, et al	Cancer Research	2004	4660	245.26
2	A national cancer institute workshop on microsatellite instability for cancer detection and familial predisposition: development of international criteria for the determination of microsatellite instability in colorectal cancer	Boland CR, et al	Cancer Research	1998	3353	134.12
3	Leucovorin and fluorouracil with or without oxaliplatin as first-line treatment in advanced colorectal cancer	Gramont A, et al	Journal of Clinical Oncology	2000	2997	130.3
4	Clinical score for predicting recurrence after hepatic resection for metastatic colorectal cancer: analysis of 1001 consecutive cases	Fong Y, et al	Annals of Surgery	1999	2669	111.21
5	Colorectal cancer statistics, 2017	Siegel RL, et al	CA: A Cancer Journal for Clinicians	2017	2604	434
6	Wild-type KRAS is required for panitumumab efficacy in patients with metastatic colorectal cancer	Amado RG, et al	Journal of Clinical Oncology	2008	2474	164.93
7	FOLFIRI followed by FOLFOX6 or the reverse sequence in advanced colorectal cancer: a randomized GERCOR study	Tournigand C, et al	Journal of Clinical Oncology	2004	2289	120.47
8	Bevacizumab in combination with oxaliplatin-based chemotherapy as first-line therapy in metastatic colorectal cancer: a randomized phase III study	Saltz LB, et al	Journal of Clinical Oncology	2008	2240	149.33
9	Global patterns and trends in colorectal cancer incidence and mortality	Arnold M,et al	Gut	2017	2168	361.33
10	Colorectal cancer statistics, 2014	Siegel R, et al	CA: A Cancer Journal for Clinicians	2014	2153	239.22
11	The mesorectum in rectal-cancer surgery – the clue to pelvic recurrence	Heald RJ, et al	British Journal of Surgery	1982	1880	45.85
12	Characterization of the human-colon carcinoma cell-line (caco-2) as a model system for intestinal epithelial permeability	Hidalgo IJ, et al	Gastroenterology	1989	1843	54.21
13	A Randomized controlled trial of fluorouracil plus leucovorin, irinotecan, and oxaliplatin combinations in patients with previously untreated metastatic colorectal cancer	Goldberg RM, et al	Journal of Clinical Oncology	2004	1791	94.26
14	Bevacizumab in combination with oxaliplatin, fluorouracil, and leucovorin (FOLFOX4) for previously treated metastatic colorectal cancer: results from the Eastern Cooperative Oncology Group Study E3200	Giantonio BJ, et al	Journal of Clinical Oncology	2007	1780	111.25
15	KRAS mutation status is predictive of response to cetuximab therapy in colorectal cancer	Lievre A, et al	Cancer Research	2006	1750	102.94
16	New clinical criteria for hereditary nonpolyposis colorectal cancer (HNPCC, Lynch Syndrome) proposed by the International Collaborative Group n HNPCC	Vasen HFA, et al	Gastroenterology	1999	1733	72.21
17	ESMO consensus guidelines for the management of patients with metastatic colorectal cancer	Cutsem EV, et al	Annals of Oncology	2016	1614	230.57
18	Laparoscopic surgery versus open surgery for colon cancer: short-term outcomes of a randomized trial	Bonjer HJ, et al	Lancet Oncology	2005	1548	86
19	Effects of *KRAS, BRAF*, NRAS, and PIK3CA mutations on the efficacy of cetuximab plus chemotherapy in chemotherapy-refractory metastatic colorectal cancer: a retrospective consortium analysis	Roock DW, et al	Lancet Oncology	2010	1530	117.69
20	Open-label phase III trial of panitumumab plus best supportive care compared with best supportive care alone in patients with chemotherapy-refractory metastatic colorectal cancer	Cutsem EV, et al	Journal of Clinical Oncology	2007	1502	93.88
21	Improved overall survival with oxaliplatin, fluorouracil, and leucovorin as adjuvant treatment in stage II or III colon cancer in the MOSAIC trial	Andre T, et al	Journal of Clinical Oncology	2009	1492	106.57
22	MicroRNA-21 (miR-21) post-transcriptionally downregulates tumor suppressor Pdcd4 and stimulates invasion, intravasation and metastasis in colorectal cancer	Asangani IA, et al	Oncogene	2008	1455	97
23	Phase II trial of cetuximab in patients with refractory colorectal cancer that expresses the epidermal growth factor receptor	Saltz LB, et al	Journal of Clinical Oncology	2004	1385	72.89
24	Cetuximab plus irinotecan, fluorouracil, and leucovorin as first-line treatment for metastatic colorectal cancer: updated analysis of overall survival according to tumor KRAS and *BRAF* mutation status	Cutsem EV, et al	Journal of Clinical Oncology	2011	1374	114.5
25	Randomized trial of cytoreduction and hyperthermic intraperitoneal chemotherapy versus systemic chemotherapy and palliative surgery in patients with peritoneal carcinomatosis of colorectal cancer	Verwaal VJ, et al	Journal of Clinical Oncology	2003	1369	68.45

AC = average citations per year, PY = publication year, TC = total citation.

### 3.9. Co-citation analysis

The references section of the 64,774 articles published on CRC cited 665,322 studies. Of these studies, the top 10 studies with the highest number of co-citations (more than 1156 citations) were those of Sauer et al (2004) (Number of citation: NC = 2486), Kapiteijn et al (2001) (NC = 1660), Bray et al (2018) (NC = 1643), Hurwitz et al (2004) (NC = 1639), Fearon et al (1990) (NC = 1583), Jemal et al (2011) (NC = 1410), Kaplan et al (1958) (NC = 1306), Gramont et al (2000) (NC = 1282), Douillard et al (2000) (NC = 1194), and Bosset et al (2006) (NC = 1156).^[16–25]^

### 3.10. Trend topics

**Figure 4.**
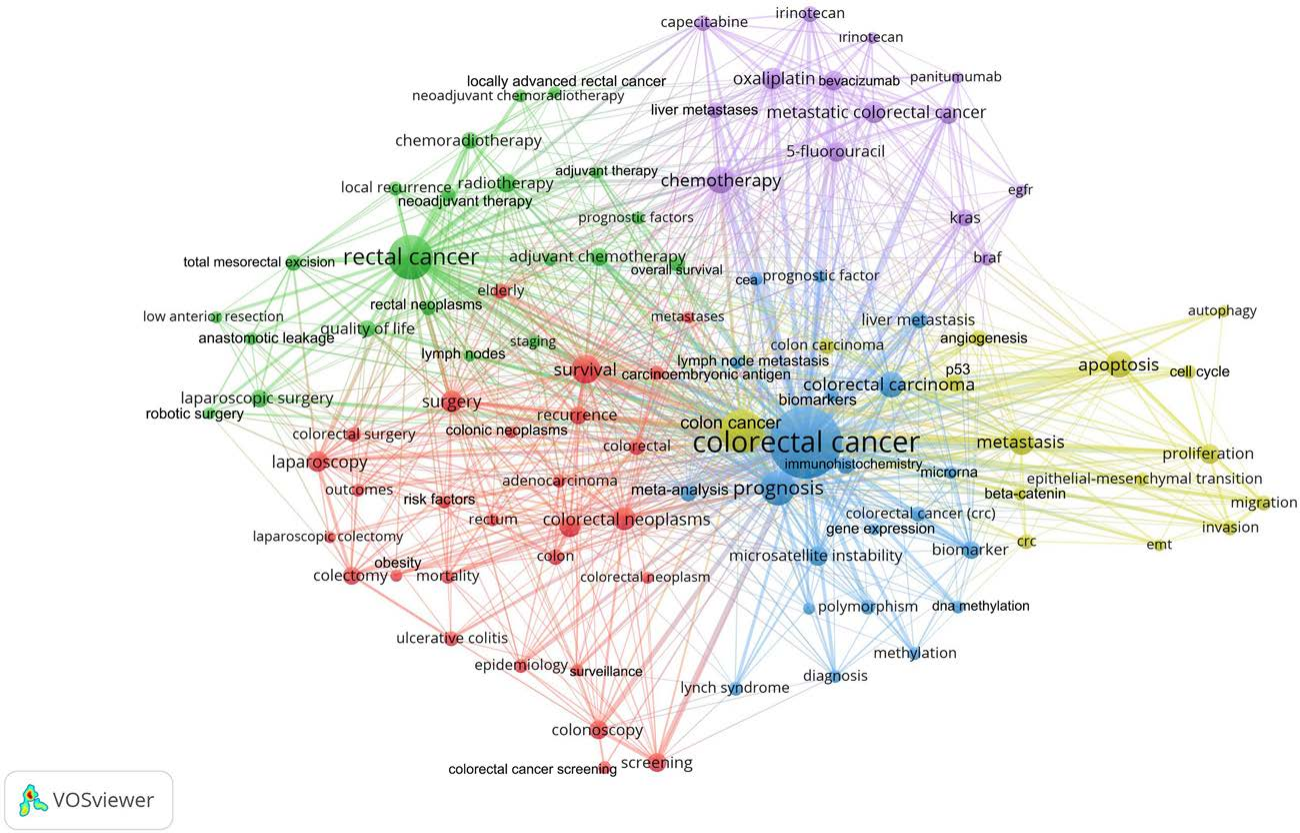
Network visualization map for cluster analysis based on keyword analysis identifying clustering of colorectal Cancer topics. Different colors indicate different clusters. Keywords in the same cluster are of the same color. The size of the circle indicates the number of uses of the keyword.

**Figure 5.**
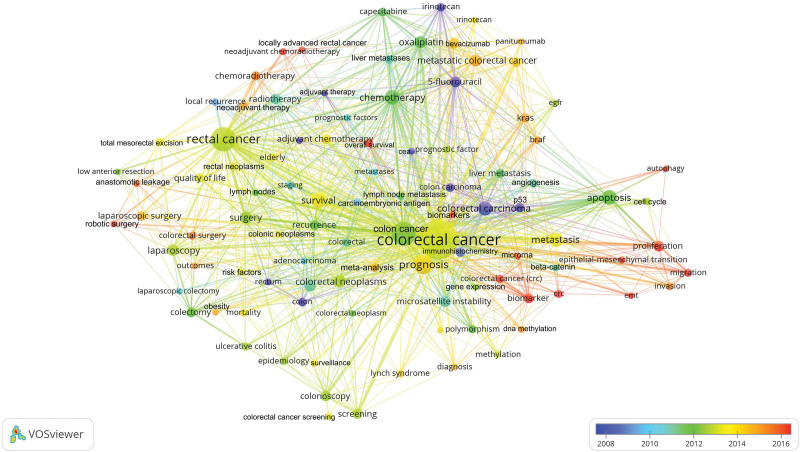
Network visualization map based on keyword analysis showing past and current trends in colorectal cancer research. In the indicator given in the lower right corner of the figure, the actuality of the topics increases from blue to red (blue-green-yellow-red). The size of the circle indicates the number of times the keyword has been used.

**Figure 6.**
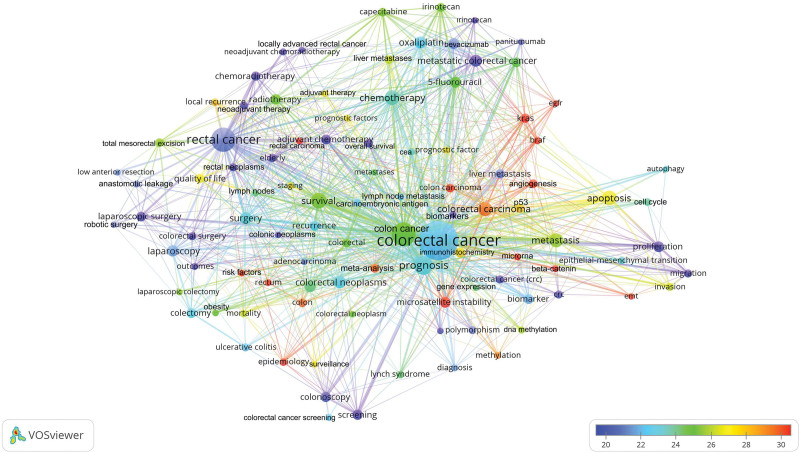
Network visualization map based on keyword analysis showing the most cited topics in colorectal cancer. In the indicator given in the lower right corner of the figure, the number of references received by the topic increases from blue to red (blue-green-yellow-red). The size of the circle indicates the number of times the keyword was used.

The 64,774 articles published on CRC used 47,496 keywords. Of these keywords, 97 had been used in at least 200 different articles, as shown in Table 3. The cluster network visualization map showing the results of the cluster analysis among these keywords is shown in Figure 4. The trend network visualization map showing trend topics is shown in Figure 5. The citation network visualization map showing the most cited topics is shown in Figure 6.

**Table 3:** The 97 most frequently used keywords in articles on colorectal cancer.

Keywords	Number of uses	Keywords	Number of uses	Keywords	Number of uses
colorectal cancer	17,904	*KRAS*	502	methylation	272
rectal cancer	5426	chemoradiotherapy	493	prognostic factor	266
colon cancer	5239	colorectal	446	biomarkers	264
prognosis	2899	capecitabine	440	diagnosis	264
survival	1807	total mesorectal excision	439	colorectal cancer screening	263
chemotherapy	1557	colon	433	outcomes	258
apoptosis	1518	liver metastases	430	rectum	257
colorectal carcinoma	1365	meta-analysis	430	adenocarcinoma	256
metastasis	1353	overall survival	411	dna methylation	255
colorectal neoplasms	1055	colon carcinoma	407	epithelial-mesenchymal transition	255
surgery	981	angiogenesis	406	CEA	253
metastatic colorectal cancer	916	rectal neoplasms	390	neoadjuvant chemoradiotherapy	253
oxaliplatin	885	invasion	383	risk factors	253
laparoscopy	866	colorectal surgery	375	prognostic factors	246
immunohistochemistry	809	migration	369	staging	243
cancer	787	colorectal cancer (CRC)	355	anastomotic leakage	242
5-fluorouracil	766	ulcerative colitis	354	EGFR	233
microsatellite instability	703	polymorphism	349	low anterior resection	233
proliferation	703	elderly	348	autophagy	230
bevacizumab	698	CRC	341	laparoscopic colectomy	220
radiotherapy	685	local recurrence	338	locally advanced rectal cancer	218
screening	664	mortality	329	panitumumab	217
colonoscopy	659	epidemiology	326	lymph nodes	216
irinotecan	643	*BRAF*	319	EMT	214
biomarker	600	lynch syndrome	312	obesity	214
recurrence	595	neoadjuvant therapy	309	robotic surgery	213
laparoscopic surgery	583	carcinoembryonic antigen	301	colonic neoplasms	211
colectomy	577	cell cycle	288	colorectal neoplasm	208
adjuvant chemotherapy	560	rectal carcinoma	282	surveillance	207
cetuximab	554	microrna	281	metastases	206
liver metastasis	530	lymph node metastasis	279	adjuvant therapy	202
P53	529	beta-catenin	272	gene expression	202
quality of life	524				

EMT = epithelial-mesenchymal transition.

## 4. Discussion

The analysis of the distribution of articles on CRC for the period of 1980 to 2021 showed that an average of 470 articles (min–max: 173–956) had been published during 1980 to 1998. It was observed that an average of 1372 articles (min–max: 1038–1873) had been published from 1999 to 2008. On the other hand, an average of 2446 articles (min–max: 2104–2777) and 3735 articles (min–max: 3218–4460) were published during 2009 to 2013 and 2014 to 2021, respectively. An increasing trend of publications began during 2007 to 2008 and continued until 2021, resulting in 4460 articles in 2021. The projected estimations for the next 5 years points to a continued upward trend of the number of articles likely to be published on CRC.

Analysis of the distribution of publications by countries found that 18 of the top 20 countries that contributed the most to the literature on CRC were the developed countries (the USA, Japan, the UK, Italy, Germany, France, South Korea, the Netherlands, Spain, Canada, Australia, Sweden, Taiwan, Denmark, Switzerland, Belgium, Norway, and Greece). Only two of the 20 most active countries in terms of publication on CRC (China, Turkey) were the developing countries. However, these countries have large economies. In the most active countries in term of CRC research, we observed the presence of strong–moderate correlations of the number of articles produced by countries on CRC with GDP, GDP per capita, and HDI, suggesting that publication productivity in the field of CRC is primarily influenced by the economy size and development status of the countries. The incidence and mortality rates of CRC are known to be associated with the level of development of the countries, especially with HDI.^[7,9]^ Our study found that the academic productivity has a high level of correlation with HDI, suggesting that increased incidence rate of CRC in developed countries leads to increased academic productivity.

The density map created while considering the score of total collaboration among countries showed that the countries with the most intensive collaboration were the USA, England (in the UK), Germany, Italy, France, Spain, the Netherlands, Australia, Sweden, Belgium, China, Canada, Japan, Denmark, Switzerland, Norway, Greece, Finland, South Korea, and Austria. Analysis of co-authorship collaboration for CRC publications among countries showed that geographical distance was a major factor in the production of articles. The groups of countries that collaborated more often are mentioned as follows: Brazil, Chile, Mexico, Argentina, and Uruguay; Algeria, Lebanon Tunisia, Libya, Cyprus, Egypt, Morocco, and South Africa; Bulgaria, France, Hungary, Poland, Portugal, Romania, Slovakia, Slovenia, Serbia, Finland, Croatia, Czech Republic, Latvia, Lithuania, Estonia, and Turkey; Ireland and North Ireland; the United Arab Emirates, Qatar, Saudi Arabia, Iran, Kuwait, Pakistan, Iraq, Oman, and Jordan; Kazakhstan, Russia, and Ukraine; Bangladesh and India; Indonesia, Japan, Malaysia, Nigeria, China, Philippines, Singapore, South Korea, Sri Lanka, Taiwan, Thailand, and Vietnam; Colombia, Denmark, Greece, Iceland, Netherlands, Norway, Spain, and Sweden; Austria, Belgium, Germany, Italy, Luxembourg, and Switzerland; England, Scotland, and Wales; and Australia and New Zealand.

The journals that published the highest number of articles on CRC were the Diseases of the Colon and Rectum, International Journal of Cancer, Anticancer Research, British Journal of Cancer, International Journal of Colorectal Disease, Cancer Research, Cancer, Oncotarget, BMC Cancer, World Journal of Gastroenterology, Colorectal Disease, Oncology Reports, Oncology Letters, Annals of Surgical Oncology, Clinical Cancer Research, Journal of Clinical Oncology, International Journal of Oncology, European Journal of Cancer, Surgical Endoscopy and Other Interventional Techniques, and Journal of Surgical Oncology (in descending order). We recommend that the authors who are in the process of doing research on CRC and wishing to publish their research should consider these journals. Analysis of the citations received by the journals found that the most influential journals based on the average number of citations per article were the following in descending order: CA-A Cancer Journal For Clinicians, Lancet Oncology, Journal of Clinical Oncology, Cancer and Metastasis Reviews, Cancer Discovery, Cancer Cell, APC Proteins, Journal of the National Cancer Institute, Bone Marrow Transplantation, Gastroenterology, Inflammation and Cancer, Journal of the National Cancer Institute, Annals of Surgery, Cancer Research, Cancer Genetics, Oncogene Research, Nature Reviews Cancer, Journal of Thoracic and Cardiovascular Surgery, Journal of Neurology Neurosurgery and Psychiatry, and Gut. We recommend that researchers aiming to maximize the number of citations received by their articles should primarily consider these journals.

Analysis of the articles based on the total number of citations found that the most cited study was the study by Andersen et al (2004) titled “Normalization of real-time quantitative reverse transcription-PCR data: A model-based variance estimation approach to identify genes suited for normalization, applied to bladder and colon cancer data sets” published in Cancer Research.^[26]^ The second most influential study was the study published by Boland et al entitled “A national cancer institute workshop on microsatellite instability for cancer detection and familial predisposition: development of international criteria for the determination of microsatellite instability in colorectal cancer” published in Cancer Research.^[27]^ The third most influential study was published by de Gramont et al^[23]^ that was entitled “Leucovorin and fluorouracil with or without oxaliplatin as first-line treatment in advanced colorectal cancer” published in the Journal of Clinical Oncology. The fourth most influential study was the article published by Fong et al^[28]^ titled “Clinical score for predicting recurrence after hepatic resection for metastatic colorectal cancer: Analysis of 1001 consecutive cases” published in the Annals of Surgery. The fifth most influential study was an article by Siegel et al^[29]^ Based on the average number of citations received per year, the first and second most influential studies were the articles published by Siegel et al and Siegel et al on colorectal cancer statistics.^[6,29]^ The third most influential study was conducted by Arnold et al^[30]^ entitled “Global patterns and trends in colorectal cancer incidence and mortality” published in the GUT journal. The fourth most influential study was an article by Andersen et al.^[26]^ The fifth most influential study was Siegel et al^[31]^ article entitled “Colorectal cancer statistics, 2014” published in the CA: A Cancer Journal for Clinicians. Based on the number of co-citations of all the analyzed articles, the most influential studies were those by Sauer et al, Kapiteijn et al, Bray et al, Hurwitz et al, Fearon et al.^[16–20]^ We can recommend that clinicians and researchers interested in this topic should read these publications.

Based on the results of keyword analysis, the clustering analysis formed a cluster on CRC in five different colors (Colors for Cluster 1, red; Cluster 2, green; Cluster 3, blue; Cluster 4, yellow; Cluster 5, purple). The most cited keywords were *KRAS, BRAF*, estimated glomerular filtration rate, colon carcinoma, angiogenesis, microsatellite instability, microRNA, beta-catenin, epithelial-mesenchymal transition (EMT), rectal carcinoma, risk factors, rectum, epidemiology, colorectal carcinoma, meta-analysis, methylation, colon, and P53. Analysis of the trend topics found that the following keywords were studied in recent years: proliferation, biomarker, migration, EMT, overall survival, autophagy, neoadjuvant chemoradiotherapy, locally advanced rectal cancer, biomarkers, robotic surgery, anastomotic leakage, chemoradiotherapy, metastatic colorectal cancer, *KRAS, BRAF*, invasion, methylation, meta-analysis, colorectal surgery, laparoscopic surgery, and neoadjuvant therapy.

Our literature review on CRC found no comprehensive bibliometric study that was directly related to CRC. The strength of our comprehensive study is that it is the first bibliometric research on this subject. Wrafter et al^[32]^ identified the top 100 articles on CRC with the highest number of citations. Darroudi et al conducted bibliometric research on CRC treatment, whereas Jin et al conducted bibliometric research on the management of liver metastasis in CRC.^[33,34]^ The literature review in this study was confined to the WoS database, which can be a limitation of the study. However, the PubMed database does not allow citation and co-citation analysis. The Scopus database, on the other hand, also indexes low-impact journals. Compared to other databases, the WoS database indexes articles published in higher-impact journals.^[11–13]^

## 5. Conclusion

This research on CRC has exhibited an upward trend in the number of articles in recent years, and this comprehensive bibliometric research provided an overview of 64,774 articles published between 1980 and 2021. We hypothesize that the number of articles on CRC will continue to grow in number. Analysis of the trend topics revealed that the following keywords were studied in recent years: proliferation, biomarker, migration, EMT, overall survival, autophagy, neoadjuvant chemoradiotherapy, locally advanced rectal cancer, biomarkers, robotic surgery, anastomotic leakage, chemoradiotherapy, metastatic colorectal cancer, *KRAS, BRAF*, invasion, methylation, meta-analysis, colorectal surgery, laparoscopic surgery, and neoadjuvant therapy. We believe that further research is needed to elucidate the causes of the increasing incidence rates of CRC in young and middle-aged adults. Although there are international collaborations occurring globally, CRC research should be further supported and increased, especially in the underdeveloped countries. This article can guide clinicians, scientists, and surgical assistants about the global outcomes of research on CRC.

## Author contributions

**Conceptualization:** İbrahim Tayfun Şahiner, Çetin Altunal.

**Data curation:** İbrahim Tayfun Şahiner.

**Formal analysis:** İbrahim Tayfun Şahiner, Çetin Altunal.

**Investigation:** İbrahim Tayfun Şahiner, Çetin Altunal.

**Methodology:** İbrahim Tayfun Şahiner, Çetin Altunal.

**Project administration:** İbrahim Tayfun Şahiner.

**Resources:** İbrahim Tayfun Şahiner, Çetin Altunal.

**Software:** İbrahim Tayfun Şahiner.

**Supervision:** İbrahim Tayfun Şahiner.

**Validation:** İbrahim Tayfun Şahiner.

**Visualization:** İbrahim Tayfun Şahiner.

**Writing – original draft:** İbrahim Tayfun Şahiner, Çetin Altunal.

**Writing – review & editing:** İbrahim Tayfun Şahiner, Çetin Altunal.
